# Higher Risk for Sjögren’s Syndrome in Patients With Fibromyalgia: A Nationwide Population-Based Cohort Study

**DOI:** 10.3389/fimmu.2021.640618

**Published:** 2021-04-12

**Authors:** Shuo-Yan Gau, Pui-Ying Leong, Cheng-Li Lin, Hsi-Kai Tsou, James Cheng-Chung Wei

**Affiliations:** ^1^ School of Medicine, Chung Shan Medical University, Taichung, Taiwan; ^2^ Division of Allergy, Immunology and Rheumatology, Department of Internal Medicine, Chung Shan Medical University Hospital, Taichung, Taiwan; ^3^ Institute of Medicine, Chung Shan Medical University, Taichung, Taiwan; ^4^ Management Office for Health Data, China Medical University Hospital, Taichung, Taiwan; ^5^ College of Medicine, China Medical University, Taichung, Taiwan; ^6^ Functional Neurosurgery Division, Neurological Institute, Taichung Veterans General Hospital, Taichung, Taiwan; ^7^ Department of Rehabilitation, Jen-Teh Junior College of Medicine, Nursing and Management, Houlong, Taiwan; ^8^ College of Health, National Taichung University of Science and Technology, Taichung, Taiwan; ^9^ Graduate Institute of Integrated Medicine, China Medical University, Taichung, Taiwan

**Keywords:** fibromyalgia, cohort, Sjögren’s syndrome, population based study, NHIRD

## Abstract

**Objectives:**

Clinically, associations have been observed between Sjögren’s syndrome and fibromyalgia. Nonetheless, population-based evidence evaluating the risk of Sjögren’s syndrome in fibromyalgia patients is lacking. The main purpose of this retrospective cohort study was to determine the association between fibromyalgia and subsequent development of Sjögren’s syndrome.

**Methods:**

This retrospective cohort study extracted data from the Longitudinal Health Insurance Database (LHID) of the Taiwan National Health Insurance (NHI). During 2000-2012, patients with newly-diagnosed fibromyalgia (International Classification of Diseases, Ninth Revision, Clinical Modification code 729.1) were defined as the exposure cohort. Age- and gender-matched individuals without fibromyalgia were used as the comparison cohort. The adjusted hazard ratios (aHR) for the occurrence of Sjögren’s syndrome in those with fibromyalgia were evaluated along with stratified analyses of different subgroups.

**Results:**

Of the 149,706 subjects whose data were extracted from the LHID, 74,853 subjects had coded fibromyalgia and 74,853 control subjects were without fibromyalgia. Compared to the control group, patients with fibromyalgia had an aHR of 2.00 (95% Confidence Interval [CI], 1.52-2.61) for developing Sjögren’s syndrome. In fibromyalgia patients aged 20-49 years, the aHR for future Sjögren’s syndrome was 3.07 (95% CI, 1.92-4.89).

**Conclusion:**

Patients with fibromyalgia, both males and females, have a higher risk for developing Sjögren’s syndrome than those without fibromyalgia, especially those aged 20-49 years. While managing patients, clinicians should be aware of the bidirectional association between the two diseases, which helps to understand the impact of the association on disease activity and diagnosis.

## Introduction

Sjögren’s syndrome is caused by inflammation of the exocrine glands. In response to inflammation of glandular cells, lymphocytes of the HLA-DR immune system infiltrate the glands, which leads to cell destruction and an imbalance of cytokines. Glandular cell destruction and impaired secreting function together produce symptoms such as pain, fatigue, xerophthalmia or mucosal surface dryness (1–3). Epigenetic dysregulation and environmental factors, including smoking or viral infections, serve as etiological factors in Sjögren’s syndrome (1, 4). Pain in Sjögren’s syndrome is attributed to nociceptive and neuropathic factors, and pain-related diseases such as fibromyalgia are prevalent in patients with Sjögren’s syndrome (2, 5).

Fibromyalgia typically causes multifocal and migratory pain, and may be accompanied by comorbid symptoms associated with the central nervous system, particularly fatigue and sleep disturbances (6). Since the adopted diagnostic criteria of fibromyalgia vary between different geographic regions, prevalence of fibromyalgia has been difficult to identify. Häuser et al. (7) reported that the prevalence of fibromyalgia ranged from 2-4% in epidemiological studies. In Asia, the overall prevalence of fibromyalgia varies from 0.8% to 8.8%, whereas in Europe, the prevalence varies from 0.4% to 5.4%. The occurrence of fibromyalgia will influence patients’ cytokine levels. For instance, T helper 2 (Th2) anti-inflammatory cytokines, including IL-4, IL-5 and IL-13, have been reported to decrease in fibromyalgia patients, whereas inflammatory cytokines IL-6 and IL-8 are found to elevate in fibromyalgia, indicating a potential role in the chronic pain of fibromyalgia (8, 9). Fibromyalgia prevalence is also reported to be high in autoimmune diseases such as ankylosing spondylitis and rheumatoid arthritis. Such co-existence of fibromyalgia potentially affects the severity of autoimmune diseases (10, 11).

Considering the common symptoms shared by fibromyalgia and Sjögren’s syndrome—including fatigue, pain, sicca and xerostomia—the association between the two has long been discussed (2, 12, 13). Comorbidities of the two diseases overlap widely (14), and previous research has suggested that Sjögren’s syndrome may play a role in the pathophysiology of fibromyalgia (12). Clinically, the symptoms of the two diseases also overlap widely. However, though the incidence of fibromyalgia in patients with Sjögren’s syndrome has been reported, to the best of our knowledge, large-scale studies evaluating the association between fibromyalgia and future Sjögren’s syndrome are lacking. Therefore, the objectives of this cohort study were to evaluate whether fibromyalgia is associated with higher risk for subsequent Sjögren’s syndrome and to further evaluate the risk of Sjögren’s syndrome between different age and sex subgroups using a Taiwanese population-based database.

## Materials and Methods

### Data Source

All data analyzed in the present study were extracted from the Longitudinal Health Insurance Database (LHID), a national repository of health insurance claims. In 1995, the Taiwan government launched the National Health Insurance (NHI) program, which ensures healthcare access for more than 99% of the nation’s residents. All medical claims such as inpatient visits, admission records and therapy are collected and stored in the NHI Research Database (NHIRD). The LHID, a subset of the NHIRD containing one million randomly-selected patients from the NHI, was utilized for all data analysis in the present study. The distribution of age and gender in the LHID is similar to that in the Taiwanese population.

The diagnostic codes follow the International Classification of Diseases, Ninth Revision, Clinical Modification (ICD-9-CM). Individual identification numbers were encrypted to protect the personal privacy of subjects. This study conformed to the principles of the Declaration of Helsinki and was approved by the Institutional Review Board of China Medical University Hospital [CMUH-REC2-115(CR-4)].

### Study Population

This cohort study included a case cohort and a control cohort. Patients with at least two ambulatory visits or one hospital admission for fibromyalgia (ICD-9-CM code 729.1) from 2000 to 2012 were included as subjects in the case cohort. To audit the authenticity of fibromyalgia diagnosis, these patients also should have been treated with fluoxetine, duloxetine, milnacipran, pregabalin, amitriptyline or tramadol after the diagnosis of fibromyalgia. Patients never diagnosed with fibromyalgia served as the control subjects in this study. The index date for patients in the case cohort was the date of the first diagnosis of fibromyalgia; that of patients in the control cohort was a random date between 2000 and 2012. The exclusion criteria were: aged less than 18 years old and diagnosis of Sjögren’s syndrome before the index date. One control subject was matched by age, gender and index year to each case subject. All included patients were observed from the index date to the endpoint of this study (December 31, 2013), death or withdrawal from the NHI, whichever came first.

### Main Outcome and Covariates

The primary endpoint of this study was the diagnosis of Sjögren’s syndrome (ICD-9-CM code 710.2). The catastrophic illness certificate (CIC) is a requirement in Taiwan for additional benefits associated with a catastrophic illness. In Taiwan, if a patient would like to apply for a CIC, related clinical and laboratory information (including physical examinations, autoantibodies, lip biopsy and salivary scintigraphy, etc.) must be provided by rheumatologists. For patients with Sjögren’s syndrome, all submitted information was assessed by a review committee in accordance with the criteria of the American-European Consensus Group (15). Previous studies also utilized the CIC for defining Sjögren’s syndrome (16, 17). Therefore, as an additional criterion, we confirmed that subjects had developed Sjögren’s syndrome if they had a CIC for Sjögren’s syndrome. The following comorbidities were considered to be potentially confounding: chronic urticaria (ICD-9-CM code 708), inflammatory bowel disease (ICD-9-CM code 555, 556), hyperlipidemia (ICD-9-CM code 272), chronic obstructive pulmonary disease (ICD-9-CM code 490–496), osteoporosis (ICD-9-CM code 733.0), chronic kidney disease (ICD-9-CM code 585), rheumatoid arthritis (ICD-9-CM code 714.0), ankylosing spondylitis (ICD-9-CM code 720), sleep disorder (ICD-9-CM codes 307.4, 780.5), hepatitis C (ICD-9-CM code 070.7) and depression (ICD-9-CM codes 296.2, 296.3, 296.82, 300.4, 311). Subjects who received more than 90 days of systemic steroids or Non-Steroidal Anti-Inflammatory Drugs (NSAIDs) as systemic steroid users and NSAID users, respectively.

### Statistical Analysis

To compare the demographic characteristics, comorbidities and medications of the case cohort and the control cohort, the chi-square test was applied for categorical variables and Student t-test for continuous variables (i.e., mean age). The univariable Cox proportional hazard model was utilized to estimate the hazard ratio (HR) and 95% confidence interval (CI). HRs were adjusted by including variables found significant in the univariable model into the multivariable model. The cumulative incidence curves for Sjögren’s syndrome were obtained using the Kaplan-Meier method and examined using the log-rank test. Data analyses were carried out using SAS software (version 9.4 for Windows; SAS Institute, Inc., Cary, NC, USA). A p-value of less than 0.05 was the statistical significance threshold.

### Role of the Funding Source

The Funders helped this study with access to the database and technique support in data analysis.

## Results

### Baseline Characteristics

A total of 149,706 subjects [74,853 fibromyalgia (case) patients and 74,853 non-fibromyalgia control patients] were included in this cohort study. The mean follow-up time for the case cohort was 6.88 (± 3.97) years. After matching by age and gender, patients in the case cohort (51.2 ± 16.3 years) had a slightly older mean age than patients in the control cohort (50.6 ± 16.7 years), see **Table 1**. The proportions of male and female patients were the same in both cohorts. More patients with fibromyalgia in the case cohort had comorbidities and took medication compared to patients without fibromyalgia in the control cohort.

**Table 1:** Baseline characteristics of individuals with and without fibromyalgia.

	Fibromyalgia				*p*-value
	No		Yes		
	N = 74,853		N = 74,853		
	n	%	n	%	
**Age (years)**					0.99
20−49	36,325	48.5	36,325	48.5	
50−64	21,199	28.3	21,199	28.3	
≥ 65	17,329	23.2	17,329	23.2	
Mean (SD)^⁑^	50.6	16.7	51.2	16.3	0.0001
**Gender**					0.99
Women	44,796	59.9	44,796	59.9	
Men	30,057	40.2	30,057	40.2	
**Comorbidity**					
Chronic urticaria	7,271	9.71	12,884	17.2	<0.001
Inflammatory bowel disease	980	1.31	1,880	2.51	<0.001
Hyperlipidemia	12,914	17.3	22,067	29.5	<0.001
Chronic obstructive pulmonary disease	12,883	17.2	21,929	29.3	<0.001
Osteoporosis	4,949	6.61	8,947	12.0	<0.001
Chronic kidney disease	1,184	1.58	1,734	2.32	<0.001
Rheumatoid arthritis	121	0.16	179	0.24	<0.001
Ankylosing spondylitis	256	0.34	629	0.84	<0.001
Hepatitis C	755	1.01	1,419	1.90	<0.001
Depression	2,651	3.54	8,336	11.1	<0.001
Sleep disorder	10,413	13.9	24,392	32.6	<0.001
**Medications**					
Systemic Steroids	5,926	7.92	15,458	20.7	<0.001
NSAIDs	16,958	22.7	32,042	42.8	<0.001

Chi-square test; ^⁑^t-test.

SD, standard deviation; NSAIDs, non-steroidal anti-inflammatory drugs.

### Incidence and Hazard Ratios of Sjögren’s Syndrome

**Figure 1:**
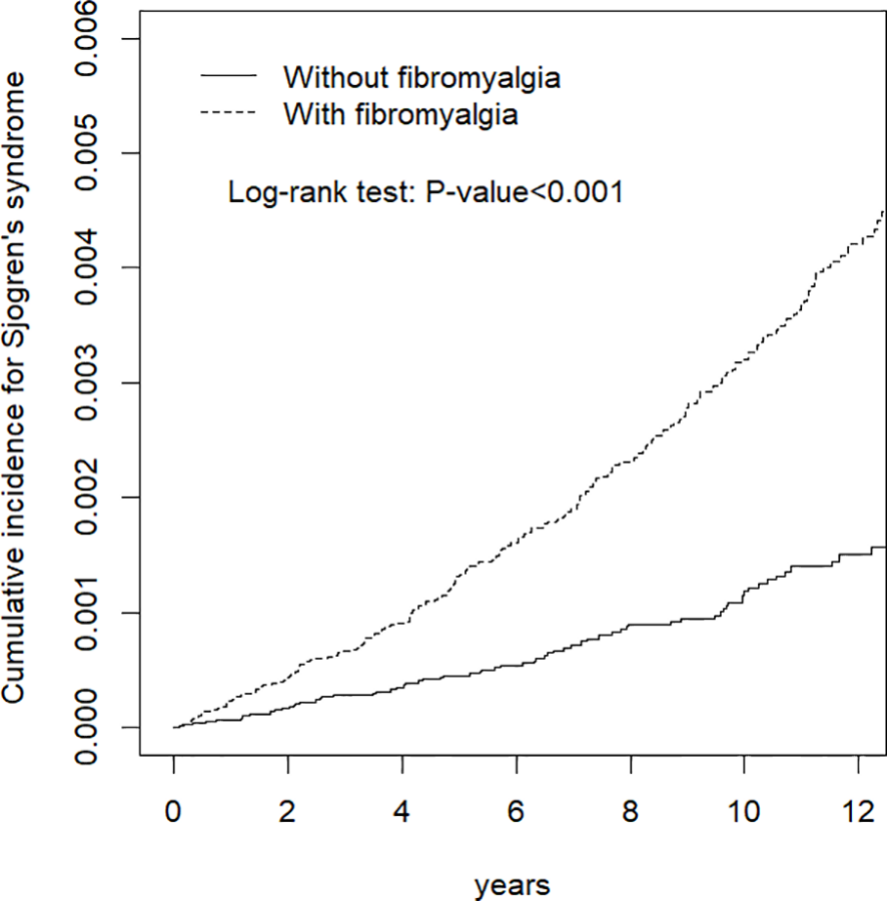
Cumulative incidence of Sjögren’s syndrome in adults with and without fibromyalgia.

As shown in **Figure 1**, the cumulative incidence curve for Sjögren’s syndrome in fibromyalgia patients was significantly higher than that for non-fibromyalgia patients (log-rank: p-value <0.01). **Table 2** demonstrates the incidence rate and HR for Sjögren’s syndrome. The incidence rates of Sjögren’s syndrome in patients with and without fibromyalgia were 3.39 and 1.24 per 10,000 person-years, respectively. After adjustment, patients with fibromyalgia had double the risk of Sjögren’s syndrome compared to controls (adjusted HR [aHR] 2.00, 95% CI 1.52, 2.61). The relationship between age and Sjögren’s syndrome was not significant after controlling for gender and comorbidities. Compared to men, the risk for Sjögren’s syndrome in women was 3.56 times (95% CI 2.53, 5.01) higher. Patients with chronic urticaria, osteoporosis, ankylosing spondylitis, depression or sleep disorder had an increased risk for developing Sjögren’s syndrome compared to those without these comorbidities.

**Table 2:** Incidence of and risk factors for Sjögren’s syndrome.

	Event	PY	Rate^#^	Crude HR (95% CI)	Adjusted HR ^＆^(95% CI)
**Fibromyalgia**					
No	80	644,935	1.24	1.00	1.00
Yes	226	667,611	3.39	2.69(2.09, 3.48)***	2.00(1.52, 2.61)***
**Age (years)**					
20−49	127	681,892	1.86	1.00	1.00
50−64	117	375,736	3.11	1.70(1.32, 2.18)***	1.27(0.97, 1.67)
≥ 65	62	254,918	2.43	1.40(1.03, 1.90)*	0.97(0.69, 1.37)
**Gender**					
Women	267	811,203	3.29	4.13(2.95, 5.78)***	3.56(2.53, 5.01)***
Men	39	501,343	0.78	1.00	1.00
**Comorbidity**					
Chronic urticaria					
No	243	1,158,045	2.10	1.00	1.00
Yes	63	1,545,02	4.08	2.12(1.61, 2.81)***	1.56(1.18, 2.07)**
Inflammatory bowel disease					
No	300	1,290,470	2.32	1.00	1.00
Yes	6	22,076	2.72	1.26(0.56, 2.82)	
Hyperlipidemia					
No	201	1,026,456	1.96	1.00	1.00
Yes	105	286,000	3.67	1.94(1.53, 2.46)***	1.23(0.95, 1.59)
Chronic obstructive pulmonary disease					
No	210	1,038,839	2.02	1.00	1.00
Yes	96	273,707	3.51	1.84(1.45, 2.35)***	1.27(0.98, 1.65)
Osteoporosis					
No	233	1,198,625	1.94	1.00	1.00
Yes	73	113,921	6.41	3.40(2.62, 4.43)***	1.75(1.30, 2.35)***
Chronic kidney disease					
No	301	1,294,265	2.33	1.00	1.00
Yes	5	18,281	2.74	1.30(0.54, 3.15)	
Rheumatoid arthritis					
No	305	1,310,191	2.33	1.00	1.00
Yes	1	2,355	4.25	1.90(0.27, 13.5)	
Ankylosing spondylitis					
No	300	1,305,528	2.30	1.00	1.00
Yes	6	7,018	8.55	3.89(1.73, 8.72)**	3.12(1.39, 7.02)**
Hepatitis C					
No	299	1,297,558	2.30	1.00	1.00
Yes	7	14,989	4.67	2.18(1.03, 4.60)*	1.35(0.64, 2.88)
Depression					
No	260	1,228,251	2.12	1.00	1.00
Yes	46	84,295	5.46	2.74(2.00, 3.75)***	1.57(1.12, 2.18)**
Sleep disorder					
No	193	1,044,180	1.85	1.00	1.00
Yes	113	268,367	4.21	2.49(1.97, 3.14)***	1.40(1.09, 1.81)**
**Medications**					
Systemic Steroids					
No	231	1,110,496	2.08	1.00	1.00
Yes	75	202,050	3.71	1.72(1.32, 2.23)***	1.17(0.90, 1.54)
NSAIDs					
No	191	820,132	2.33	1.00	1.00
Yes	115	492,415	2.34	0.93(0.74, 1.17)	

CI, confidence interval; CKD, chronic kidney disease; CLD, chronic liver disease and cirrhosis; HR, hazard ratio; PY, person-years; NSAIDs, non-steroidal anti-inflammatory drugs.

^#^Incidence rate per 10,000 person-years.

^&^Multivariable analysis, including age, gender, and comorbidities of diabetes mellitus, CKD, CLD, rheumatic disease, stroke, cancer and malnutrition.

*p <0.05, **p <0.01, ***p <0.001.

### Stratification Analysis

The result of stratification analysis for Sjögren’s syndrome by age, gender, comorbidities and medication are shown in **Table 3**. In subjects aged 20-49 years, the risk of Sjögren’s syndrome for those with fibromyalgia increased by 3.07 times (95% CI 1.92, 4.89) over that of non-fibromyalgia controls. The HR for Sjögren’s syndrome in patients with fibromyalgia relative to those without fibromyalgia was 1.90 in females (95% CI 1.43, 2.52) and 2.88 in males (95% CI 1.23, 6.75). In patients with no comorbidities, the development of fibromyalgia raised the risk of Sjögren’s syndrome 3.37-fold (95% CI 2.00, 5.67). In those with any one comorbidity, the HR for Sjögren’s syndrome was 1.69 (95% CI 1.25, 2.28). In patients with comorbid depression, the occurrence of fibromyalgia increased risk of Sjögren’s syndrome development by 2.27-fold (95% CI 1.69, 3.04). The risk for developing Sjögren’s syndrome in patients with fibromyalgia but not receiving systemic steroids was 2.43 times (95% CI 1.79, 3.28) higher than that of patients without fibromyalgia who used systemic steroids. Whether or not patients took NSAIDs, fibromyalgia increased the risk of developing Sjögren’s syndrome.

**Table 3:** Incidence and hazard ratios of Sjögren’s syndrome for individuals with and without fibromyalgia.

	Fibromyalgia						Crude HR (95% CI)	Adjusted HR ^＆^(95% CI)
	No			Yes				
	Event	PY	Rate^#^	Event	PY	Rate^#^		
Age								
20−49	24	339053	0.71	103	342839	3.00	4.23(2.71, 6.60)***	3.07(1.92, 4.89)***
50−64	34	186001	1.83	83	189735	4.37	2.36(1.58, 3.52)***	1.50(0.98, 2.30)
≥ 65	22	119881	1.84	40	135037	2.96	1.56(0.92, 2.62)	1.49(0.87, 2.57)
Gender								
Women	73	398968	1.83	194	412235	4.71	2.54(1.94, 3.32)***	1.90(1.43, 2.52)***
Men	7	245967	0.28	32	255377	1.25	4.35(1.92, 9.85)***	2.88(1.23, 6.75)***
Comorbidity^§^								
No	22	391194	0.56	46	223965	2.05	3.43(2.06, 5.72)***	3.37(2.00, 5.67)***
Yes	58	253741	2.29	180	443646	4.06	1.72(1.28, 2.32)***	1.69(1.25, 2.28)***
Depression								
No	70	626381	1.12	190	601871	3.16	2.77(2.11, 3.65)***	2.27(1.69, 3.04)***
Yes	10	18554	5.39	36	65741	5.48	0.97(0.48, 1.96)	0.78(0.38, 1.61)
Medications								
Systemic Steroids								
No	63	592000	1.06	168	518496	3.24	3.03(2.27, 4.06)***	2.43(1.79, 3.28)***
Yes	17	52935	3.21	58	149116	3.89	1.14(0.67, 1.96)	0.83(0.47, 1.45)
NSAIDs								
No	59	475679	1.24	132	344453	3.83	3.15(2.32, 4.28)***	1.93(1.37, 2.71)***
Yes	21	169256	1.24	94	323158	2.91	2.28(1.42, 3.67)***	2.24(1.38, 3.64)***

CI, confidence interval; HR, hazard ratio; PY, person-years.

^#^Incidence rate per 10,000 person-years.

^&^Multivariable analysis including age, gender, and comorbidities of diabetes mellitus, CKD, CLD, rheumatic disease, stroke, cancer, and malnutrition.

^§^Individuals with any comorbidity of diabetes mellitus, CKD, CLD, rheumatic disease, stroke, cancer, and malnutrition were classified into the comorbidity group.

***p <0.001.

## Discussion

The present study is the first population-based cohort study to evaluate the subsequent risk for Sjögren’s syndrome in adults with fibromyalgia. Results show that patients with fibromyalgia have a higher risk for developing Sjögren’s syndrome, especially those aged 20-49 years.

Studies have indicated a high prevalence of fibromyalgia in patients with Sjögren’s syndrome (5, 18). Chronic pain or psychological factors are associated with neuroinflammation, which triggers immune cells in the central nervous system and thus affects the serum levels of cytokines. Neuroinflammation is a critical mechanism in the pathogenesis of fibromyalgia (19). Previous studies have observed systemic activation of T lymphocytes and the subsequent imbalance in pro-inflammatory cytokines such as T helper 17 (Th-17) in patients with fibromyalgia (20, 21). Immune activation of different tissues is the main trigger for the occurrence of Sjögren’s syndrome (1). Disturbances in the pro-inflammatory cytokines also play a critical role in the pathogenesis of Sjögren’s syndrome. Peripherally elevated Th-17 cell activity, with the co-expression of interleukin 17 and interferon gamma, is associated with chronic inflammation characteristic of Sjögren’s syndrome (22). Additionally, fibromyalgia and Sjögren’s syndrome share common symptomatic features such as fatigue and stress. In fibromyalgia patients, decreased activity of the hypothalamic–pituitary–adrenal (HPA) axis has been reported (23, 24). Neuroendocrine abnormalities are also commonly present in patients with Sjögren’s syndrome. Research has indicated that hypo-responsiveness of the HPA axis, influencing the neuroendocrine system, contributes to the fatigue characteristic of Sjögren’s syndrome (25, 26). Both fibromyalgia and Sjögren’s syndrome are associated with altered serum levels of cytokines, and share common risk factors such as insomnia (27, 28). Additionally, the chronic fatigue and pain caused by fibromyalgia may lead to dysfunction of the HPA axis, which also explains abnormalities in the neuroendocrine system in Sjögren’s syndrome patients. Finally, in a retrospective study, one-third of fibromyalgia patients tested positive for Sjögren’s syndrome autoantibodies (12). Nonetheless, the relationship between fibromyalgia and the development of Sjögren’s syndrome remains unclear. The present study takes a first step in characterizing that relationship by showing that adults with fibromyalgia have a greater risk for subsequent development of Sjögren’s syndrome.

Studies have reported the interplay between depression, fibromyalgia and Sjögren’s syndrome, and the co-existence of these disease has been widely observed (29, 30). In accord with this generally known association, the present study showed that patients with depression were more likely to have Sjogren’s syndrome (**Table 2**). According to the results of stratification analysis, people with co-existing fibromyalgia and depression showed a non-significant risk of developing Sjögren’s syndrome, and in patients with no depression, the development of fibromyalgia increased the risk of Sjögren’s syndrome by 2.27-fold. This result strengthened our conclusion that fibromyalgia significantly increases the risk of developing Sjögren’s syndrome.

Females have a higher risk than males of developing fibromyalgia (6). Correspondingly, the present study had more female fibromyalgia patients than male fibromyalgia patients, and females had a risk about 3.5 times higher than males of developing Sjögren’s syndrome. This result corresponds to previous reports of Sjögren’s syndrome occurring predominantly in women (1). However, the aHR for developing Sjögren’s syndrome in males both with and without fibromyalgia was higher than that for females, implying that in fibromyalgia patients, the difference between genders may also influence the risk of Sjögren’s syndrome. However, Sjögren’s syndrome has been regarded as a disease occurring mainly in females with a female/male ratio of 10:1 (1). In one study, males with fibromyalgia showed a higher tendency for quality of life to be affected, particularly factors such as masculine identity (31). In the present study, the association between fibromyalgia and subsequent Sjögren’s syndrome was significant among patients not taking steroids rather than those taking steroids, with a more than two-fold higher risk for Sjögren’s syndrome development. For fibromyalgia patients, chronic pain and inflammation play a role in the development of Sjögren’s syndrome (12, 32). The use of steroids may be beneficial in relieving chronic inflammation, which may support results of the present study. Nevertheless, steroid-related mechanisms influencing the development of Sjögren’s syndrome have not been investigated in previous studies, and further studies are needed to further elucidate the mechanisms and potential interplay between steroid use, fibromyalgia and Sjögren’s syndrome.

## Strengths and Limitations

The primary strength of the present study is that it offers the first observational association between fibromyalgia and the risk for Sjögren’s syndrome. Previous studies investigating the relationship between these two diseases either lacked a control group or were subject to potential selection bias (12, 33, 34). However, these problems are overcome in the present study by utilizing the NHIRD to construct case-control cohorts. Nevertheless, this study has some limitations. First, the ICD-9-CM codes may not be precise enough to define the diagnosis of fibromyalgia, since these codes are classified as administrative data rather than prospective medical records. To avoid misclassification, six drugs commonly used to treat fibromyalgia were included as an extra criterion in the study design. Furthermore, previous studies confirmed the validity of using the ICD-9-CM code 729.1 as a basis for fibromyalgia diagnosis (35, 36). Second, residual confounders may exist. Some included patients may have an increased susceptibility for Sjögren’s syndrome because of confounders that could not be identified in the database, including psychological or infection status and individual biochemical data. However, we did attempt to match both cohorts by common comorbidities, comedications, related medical treatments and significant risk factors, including age and gender. Given the available risk factor information, we tried to minimize potential biases as much as possible. Future studies should focus on understanding the mechanism by which fibromyalgia affects the pathogenesis of Sjögren’s syndrome.

## Conclusion

In conclusion, both males and females with fibromyalgia are at higher risk of developing Sjögren’s syndrome than the general population without fibromyalgia, especially adults aged 20-49 years. For clinicians managing patients with either fibromyalgia or Sjögren’s syndrome, the potential association between these two diseases should be considered.

## Data Availability Statement

Datasets from the Longitudinal Health Insurance Database (LHID) 2000 were retrieved in this retrospective cohort study, and the data are available from the Taiwan National Health Insurance (NHI) Bureau. The data areis not publicly available because of legal restrictions regarding the “Personal Information Protection Act” in Taiwan. However, requests for data can be formally sent to the NHI bureau (https://dep.mohw.gov.tw/DOS/cp-2516-3591-113.html).

## Ethics Statement

This study conformed to the principles of the Declaration of Helsinki and the protocol was approved by the Institutional Review Board of China Medical University, with the IRB permit number CMUH104-REC2-115(CR-4). Individual identification numbers were encrypted to protect the personal privacy of subjects. Since data in NHIRD were de-identified, patient consent was exempted.

## Author Contributions

All authors contributed to the article and approved the submitted version. H-KT is the corresponding author. He takes responsibility for the integrity of the data and data analysis accuracy. Study conception and design: S-YG, JC-CW, C-LL, P-YL, and H-KT. Acquisition of data: JC-CW, C-LL, and H-KT. Analysis and interpretation of data: S-YG, JC-CW, C-LL, and H-KT. Writing (original draft preparation): S-YG, JC-CW, C-LL, and P-YL.

## Funding

This study is supported in part by the Taiwan Ministry of Health and Welfare Clinical Trial Center (MOHW109-TDU-B-212-114004), MOST Clinical Trial Consortium for Stroke (MOST 108-2321-B-039-003), and the Tseng-Lien Lin Foundation, Taichung, Taiwan. The funders helped this study with access to the database and technique support in data analysis.

## Conflict of Interest

The authors declare that the research was conducted in the absence of any commercial or financial relationships that could be construed as a potential conflict of interest.
